# Transgenic mice expressing constitutive active MAPKAPK5 display gender-dependent differences in exploration and activity

**DOI:** 10.1186/1744-9081-3-58

**Published:** 2007-11-12

**Authors:** Nancy Gerits, Werner Van Belle, Ugo Moens

**Affiliations:** 1 Department of Microbiology and Virology, Institute of Medical Biology, Faculty of Medicine, University of Tromsø, N-9037 Tromsø, Norway; 2 Department of Medical Genetics, University Hospital of Northern-Norway, N-9036 Tromsø, Norway

## Abstract

**Background:**

The mitogen-activated protein kinases, MAPKs for short, constitute cascades of signalling pathways involved in the regulation of several cellular processes that include cell proliferation, differentiation and motility. They also intervene in neurological processes like fear conditioning and memory. Since little remains known about the MAPK-Activated Protein Kinase, MAPKAPK5, we constructed the first MAPKAPK knockin mouse model, using a constitutive active variant of MAPKAPK5 and analyzed the resulting mice for changes in anxiety-related behaviour.

**Methods:**

We performed primary SHIRPA observations during background breeding into the C57BL/6 background and assessed the behaviour of the background-bred animals on the elevated plus maze and in the light-dark test. Our results were analyzed using Chi-square tests and homo- and heteroscedatic T-tests.

**Results:**

Female transgenic mice displayed increased amounts of head dips and open arm time on the maze, compared to littermate controls. In addition, they also explored further into the open arm on the elevated plus maze and were less active in the closed arm compared to littermate controls. Male transgenic mice displayed no differences in anxiety, but their locomotor activity increased compared to non-transgenic littermates.

**Conclusion:**

Our results revealed anxiety-related traits and locomotor differences between transgenic mice expressing constitutive active MAPKAPK5 and control littermates.

## Background

The mitogen-activated protein kinases, MAPKs for short, constitute cascades of signalling pathways involved in the regulation of several cellular processes that include cell proliferation, differentiation, apoptosis, motility and embryogenesis. MAPKs also influence the development of cancer, inflammatory processes and most important in this article, neurological processes [1-4].

The MAPK pathway exists of a four component module where a MAPK kinase kinase (MAP3K) activates a MAPK kinase (MAP2K) by phosphorylation. The latter subsequently phosphorylates and activates a MAPK. This occurs on two residues within the TXY activation motif. MAPKs can in turn phosphorylate downstream targets like transcription factors but also other kinases, referred to as MAPK-activated protein kinases, MAPKAPKs or MKs. This branch of the MAPKs consists of 5 subfamilies: the ribosomal S kinases (RSK), the mitogen- and stress activated protein kinases (MSK), the MAPK-interacting kinases (MNK), MK2 and MK3, and finally MK5.

Although the nomenclature suggests that MK2, MK3 and MK5 belong to the same branch of the MAPKAPKs, there exist considerable differences (in sequence and function) between MK2 and MK3 on one hand and MK5 on the other [3,5]. To date, little is known about MK5 its biological role. To explore the biological function of such a protein, one can either delete the gene encoding for the protein (knockout or KO strategy), or use a knockin strategy (see further) in mice. Examination of the resulting phenotypical changes can reveal more about the proteins' function. In the case of MK5, the KO mouse model, displayed either no particular phenotype or embryonic lethality, depending on which genetic background the mice were bred [6,7]. However, a recent report on a different MK5 KO model showed that MK5 deficiency enhanced DMBA-induced skin papillomas, which indicates a role for MK5 as tumour suppressor [8]. An alternative approach to study the function of a protein is to mutate the endogenous gene or to introduce an exogenous copy of the gene that carries the mutation, resulting in the expression of a dominant negative or constitutive active form of the protein. This approach is called a knockin (KI) strategy. The mutant form of the protein may perturb cellular functions and thereby allow the deduction of the normal role for the genuine protein. Currently, only four MAPK models that use the KI strategy have been described. These include the kinases B-Raf, MEKK3, PAK1 and TAK1. No such models exist for any of the known MAPKAPKs [9].

In this report we describe for the first time a KI MAPKAPK mouse model which expressed a constitutive active variant of MK5. The resulting mice, called MK5_*L*337*A *_transgenic mice, were bred onto a C57BL/6 background. With high brain expression levels for MK5 and the data from our primary SHIRPA protocol, which indicated a role in anxiety-related processes, we decided to conduct two validated anxiety tests (the elevated plus maze (EPM) and the light-dark box (LD)). Our results suggest an unexpected difference between male and female mice, as well as between transgenic (TG) and non-transgenic (NTG) mice in exploration and activity of the respective tests. Furthermore, these results indicate for the first time the involvement of a MAPKAPK in an anxiety-related context.

## Methods

### Construction of the vector

We mutated the first *Not*I site (NotI820) of pCMV-*β *(Clontech) to a *Kpn*I site using following primers : forward 5'-GCT-GCG-GAA-TGG-TAC-CCG-CGG-CCG-CAA-TTC-3' and reverse 5'-GAA-TTG-CGG-CCG-CGG-GTA-CCA-TTC-CGC-AGC-3'. To create the same site for the insert for ligation, we amplified the insert from pcDNA-HA-MK5 (described in [10]) using following primers: forward 5'-CGG-CGG-GGT-ACC-ATG-TAT-GAT-GTT-CCT-G-3' and reverse 5'-CTA-TAG-AAT-AGC-GGC-CGC-TAG-ATG-CAT-GC-3'. The *β *galactosidase fragment was cut out using restriction enzymes *Kpn*I and *Not*I and replaced with the cDNA sequence of HA-tagged MK5. The subsequent plasmid was sequenced and the leucine 337 of MK5 mutated to an alanine, using primers: forward 5'-CAG-GCG-CAT-GCC-GAG-CAG-GCG-GCA-AAC-ATG-AGG-ATC-3' and reverse 5'-GAT-CCT-CAT-GTT-TGC-CGC-CTG-CTC-GGC-ATG-CGC-CTG-3'. This single amino acid substitution has previously been shown to render MK5 constitutive active [10]. For microinjection the plasmid was linearized with *Sph*I to generate a 2.5 kb fragment. Transient transfection of *p*CMV-MK5_*L*337*A *_into PC12 and COS cells confirmed expression of the transgene by RT-PCR and immunoblotting with anti-HA and anti-MK5 antibodies.

### PCR

The resulting mice were genotyped by PCR using Jumpstart (Invitrogen). Following primers were used to specifically detect the transgene alone: forward 5'-GAG-CTG-GTT-TAG-TGA-ACC-GTC-3' and reverse 5'-CTT-TAT-CTG-TGA-ATC-CAC-GGC-CAT-TC-3'. The forward primer is complementary with the CMV promoter, and the reverse primer is complementary to MK5 sequences, allowing specific detection of the transgene. To validate the quality of genomic DNA, a genomic fragment of *β*-globin was amplified using the *β*-globin forward 5'-CCA-ATC-TGC-TCA-CAC-AGG-ATA-GAG-AGG-GCA-GG-3' and reverse 5'-CCT-TGA-GGC-TGT-CCA-AGT-GAT-TCA-GGC-CAT-CG-3'. The former led to a 1.6 kb fragment, the *β *globin primer set generated 500 bp fragments. PCR conditions were: 5 min. at 95°C; then for 35 cycles: 95° for 30 sec, 60°C for 30 sec, 72°C for 1 min; finally at 72°C for 10 min.

### Animals

The transgenic mouse project was approved by the "Forsøksdyrutvalget" (Laboratory Animal Department) and the "Sosial og Helsedirektorat (Social and Health Directorate) and performed in accordance with the Norwegian Animal Welfare Act and the European Community Council Derivative. Animals were maintained on a 12 h light/dark cycle (light on at 08:00) in a humidity, air and temperature (20–22°C) controlled environment, and tested during the light cycle. The animals were provided with food and tap water ad libitum. At weaning (21 days ± 1 day), the offsprings were earpunched and genotyped.

Mice were sacrificed with CO_2_. The F7 generation (all the same age) used for the anxiety tests was sacrificed at the same moment, whereas animals during background breeding were sacrificed over different time spans for reasons of fighting wounds, tumours, old age or place restraints. After asphyxiation, perfusion fixation was first performed with PBS, then 4% formaldehyde in PBS. Organs (liver, spleen, reproductive organs, heart, stomach, brain, lungs) were removed and stored overnight in 4% formaldehyde in PBS, and transferred to storage buffer containing 0.5% formaldehyde in 200 mM Hepes (pH 7.2) the next day. Normal tissue architecture was examined by hematoxyline-eosine staining on brain, heart and spleen slices.

### Behavioural phenotyping

A *modified primary SHIRPA protocol *was performed on the background bred animals [11]. Briefly, mice were observed during a five-minute period in a jar for spontaneous activity, body position, respiration and tremor. Then mice were transferred to the arena (a rat cage of 59.5 – 38 – 20 cm) where transfer arousal, palpebral closure, piloerection, gait, pelvic elevation, tail elevation, touch escape were monitored. We checked the following in or above the arena: trunk curl, limb grasping, visual placing, grip strength, pinna reflex, corneal reflex, toe pinch, wire manoeuvre, skin colour, lacrimation, salivation, provoked biting, negative geotaxis and vocalization.

During the light cycle, mice were transferred to the testing room 20 minutes prior to the anxiety tests. The *Elevated Plus Maze *(EPM) was constructed as previously described [12,13]. Mice (2 months old) were placed in the centre of the maze facing one of the open arms. We videorecorded each mouse for 5 minutes. During this time, the amount of entries into each arm was counted, the time spent in each arm and the centre time were measured and the amount of fecal boli was recorded.

The *Light-Dark Box *(LD), was constructed by using a rat cage (59.5 – 38 – 20 cm), spray painted black for one third of the area (walls and floor) and the floor of the 2/3 part was spray painted white [12,14]. The closed area contained a dark roof top above the dark area and a separation wall. In the middle of the wall at floor level there was an opening (7.5 – 7 cm) through which the mouse could pass on to the other compartment. Mice were placed in the middle of the white area with their backs towards the opening in the wall, and videorecorded for 10 minutes. After each individual mouse test, we removed excretion from both the maze and the LD and rinsed with water.

### Image analysis

We videorecorded each mouse for both tests with an IIDC Point Grey research camera connected to a standard personal computer running Linux 2.6.18. For the EPM we used a Medion lens able to acquire the full size of the elevated maze. For the light-dark test we relied on a Canon 25 mm television lens. Coriander version 1.03 grabbed images in full colour at a resolution of 1024 – 768 pixels and streamed them to V4L2 frame buffer at 15 frames per second. Mencoder version 1.0rcl-4.1.2 recorded and encoded the videostreams. After video acquisition, all images were cropped to the area under investigation. We removed the beginning of each videorecording where the mouse was placed on the maze. Time points where the mouse fell off the maze (n = 4NTG) were removed from the video stream. For the EPM, we used only the first 4500 frames (5 minutes). For the light-dark test, we used the first 9000 frames (10 minutes).

We aligned all movies using 4 calibration points and a standard affine texture mapping algorithm, without oversampling. For each video, we calculated the background as the modus of the video stream. Subtracting this background from each frame made it possible to create 'yes/no' images. These images reflect the mouse presence at specific time points. To measure the mouse-activity, we calculated the difference between each frame and the previous frame and limited the result to binary values.

**Figure 1:**
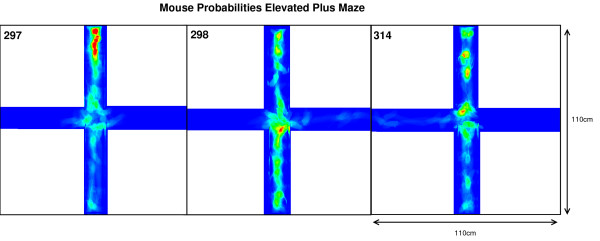
An example of the image analysis of the data per mouse on the EPM.

Once the original videorecordings were converted to mouse- and activity-videos we could create a 2 dimensional mouse-probability distribution (see Figure 1) and a 2 dimensional activity probability distribution for each mouse. The probability maps of different mouse groups were averaged and visualized using a hue colour scheme, limited to 2%, 5%, 10% and 25% for the mouse probabilities and 0.2%, 0.5%, 1% and 2% for the activity probabilities. To have a better overview of the closed versus open arms, we folded each image horizontally and vertically onto itself (see further). For both arms, profiles were generated by averaging the pixels over the width of each arm. In parallel, we compartmentalized the EPM in 7 areas: 2 areas for the outsides of the open arms (allowing us to count the nose-dips), 2 areas for the closed arms (top and bottom), 2 areas for the open arms (left and right) and 1 area for the centre area. These compartments were used to calculate the average presence and activity of the mice. The different graphs were then analyzed using a C++ program which measured the area entrances (more than 75% of the mouse volume within that area) and time fully spent in each area.

### Statistical methods

The association values between mice and the number of times they vocalized, bit or jumped were calculated using contingency table analysis by means of the algorithm given in [15]. The significance levels were calculated using Yates correction [16] since all variables could only take on two values. The probabilities that these values could be a random effect was calculated (p-value) using a standard Chi-square distribution with 1 degree of freedom. The probability that the experiment can be repeated and will give the same value was calculated (p-rep) according to [17].

The EPM and light-dark test were first analyzed as described in Methods. The numerical output was grouped into female/male transgenic/non-transgenic mice. For each group the standard deviation and mean were calculated. The difference of the means was calculated as well as the standard deviation of the difference. A one tailed T-test was performed to assess the significance of each measured difference. When the variation of the two groups was the same we relied on the homoscedastic T-test, otherwise we used the heteroscedastic T-test. We refrained from performing an ANOVA because most of the data did not show a Gausian distribution.

To avoid impact of potential differences in mouse surface area of the different groups, we calculated the mean surface area for each group and accounted for them in the further analysis. The differences in surface area were insignificant.

## Results

### Construction of the MK5_*L*337*A *_mice

**Figure 2:**
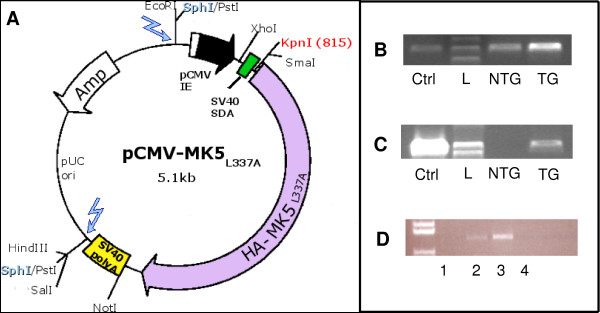
Construction of the MK5_*L*337*A *_expression plasmid for microinjection. (A) Expression vector for the delivery of the transgene. The injected fragment from the vector contains the CMV promoter, the SV40 intron, cDNA for HA-tagged MK5_*L*337*A *_and an SV40 polyA tail (see Methods). The blue arrows indicate in which restriction site (*Sph*I) the vector was linearized. Panels B to D illustrate genotyping and expression of MK5_*L*337*A *_with : (B) *β*-globin presence as a control for the DNA; (C) transgene integration as shown by PCR on genomic DNA. In panels B and C: lane 1 is the positive control (Ctrl) consisting of DNA from a TG mouse; lane 2, lkb+ ladder (L); lane 3, non-transgenic offspring, (NTG); lane 4, transgenic offspring (TG). (D) Transgenic RNA expression: in lane 1, WT C57BL/6; lane 2, mouse line A; lane 3, mouse line B transgenic offspring and lane 4, mouse line B non-transgenic littermate. The left lane in panel D represents the DNA size marker.

For the construction of the MK5_*L*337*A *_mice, 170 fertilized C57BL/6xCBA F2 eggs were injected with the linearized pCMV-MK5_*L*337*A *_fragment (2.5 kb) at the Norwegian Transgenic Centre in Oslo (see Figure 2A). From the 170 injected eggs, 47 offsprings developed to term (= 27% survival), of which 8 carried the TG (= 17%).

Positive founders and subsequent generations of transgenic mice were identified by PCR with CMV and MK5 primers as described in Materials and Methods. To assure that the lack of HA-MK5 DNA in the PCR (Figure 2B and 2C) was not the result of insufficient DNA or low DNA quality, *β*-globin primers were used to amplify *β*-globin DNA sequences. For the background breeding, we crossed three female founders with male C57BL/6 WT mice to produce the first generation of transgenic mice. Subsequent transgenic generations were bred to background with WT C57BL/6 mice for 7 generations, whereby each generation was screened for transgenic positive offsprings by PCR (Figure 2B and 2C). The expression of the transgene was verified by RNA isolation from mouse heart tissue (Figure 2D). We found the highest transgene expression in line B and decided to work with this line.

### General observations of MK5_*L*337*A *_mice during background breeding

In general, we observed no statistically significant phenotypical changes in the mice during background breeding and SHIRPA analysis. However, occasionally, the mice displayed stress reactions to handling. These reactions occurred in no immediate relationship to the transgene alone (TG vs NTG), nor to the experimentalist handling the mice. Therefore, we decided to compare male and female mice for the rest of our experiments. We started to look at differences in defensive behaviour. We observed significant increases in female transgenic (FTG) mouse vocalization, defensive biting and jump attacks compared to female non-transgenic (FNTG) mice. Male transgenic mice (MTG) mice also vocalized more, but they displayed less defensive biting and jump attacks. Despite these differences, we could not attribute any association between these parameters and the presence or the absence of the transgene (too low association values; see Table 1).

**Table 1:** Overview of defensive behaviour of mice during background breeding.

	**FNTG**	**FTG**	**MNTG**	**MTG**	**NTG**	**TG**
**Vocalization**	23	19	13	12	36	31
Population size	73	36	55	30	128	66
Percentage	31.51	52.78	23.64	40.00	28.13	46.97
TG-NTG	21.27		16.36		18.84	
Standard P	0.03		0.11		0.01	
P_rep	0.91		0.80		0.96	
Association value	0.03		0.02		0.03	

**Defensive biting**	44	23	37	17	81	40
Population size	73	36	55	30	128	66
Percentage	60.27	63.89	67.27	56.67	63.28	60.61
TG-NTG	3.61		-10.61		-2.68	
Standard P	0.72		0.33		0.72	
P_rep	0.35		0.61		0.35	
Association value	0.00		0.01		0.00	

**Jump attack**	20	14	12	3	32	17
Population size	83	45	82	49	165	94
Percentage	24.10	31.11	14.63	6.12	19.39	18.09
TG-NTG	7.01		-8.51		-1.31	
Standard P	0.39		0.14		0.80	
P_rep	0.57		0.77		0.29	
Association value	0.00		0.00		0.00	

FNTG: Female non-transgenic littermates FTG: Female transgenic mice; MNTG: Male non-transgenic littermates; MTG: Male transgenic mice. As parameters for defensive behaviour we listed vocalization, provoked biting and jump attacks. We monitored the presence (1) or absence (0) of vocalization and biting during handling and if the mice performed a jump attack (0/1). For each parameter vocalization, biting and jump attack, the number represents the number of mice that vocalized, bit or jumped, whereas the population size represents the total number of tested animals. "TG-NTG" indicates the difference between TG and NTG mice respectively. "Standard P" or the probability level *α *represents the probability that the same event happens at random and "P_rep", the likelihood that the event occurs in a new experiment. The association value represents whether the two distributions are dependent on each other.

During background breeding we also observed the following occurrences : five *Staphylococci *infections (2TG vs 3NTG), indicating no particular differences in sensitivity towards *Staphylococci *infection between TG and NTG mice. The -only- two tumours we discovered, we found in transgenic mice. Both were benign adenomas and originated from the liver and skin (results not shown). Although C57BL/6 can develop tumours spontaneously, we did not detect any in the NTG mice. We also observed at least one closed eyelid in 2 FTG, 7 FNTG mice, and 1 MNTG mouse. The C57BL/6 strain displays congenital defects in approximately 10% of the WT mice, including eye defects. The cause of the eye defect lies most likely in deficiencies in lens development. These results indicate that although the genetic background is the same for all the groups, FNTG mice are more sensitive to these eye defects than MNTG, MTG and FTG mice. In addition, we observed 4 cases of obesity (>40 g) during old age, 3 of them were founder mice and included the founder mouse for line B (which we selected for further analysis based on high TG expression levels) and 1 was an F1 TG mouse of line B. Finally, C57BL/6 mice are genetically predisposed to hydrocephaly (1–4%), we observed 2 cases in NTG mice and none in TG mice. Other C57BL/6 traits which we observed as well: high wheel activity, high incidence of tail rattling and high locomotor activity.

### Differences in open arm exploration on the EPM

Because of the preliminary results in defensive behaviour, signs of fear in the arena (tail flicks, refusal to explore) and aggression-like behaviour, we decided to perform two anxiety tests, the EPM and the LD test. Before each anxiety test, we randomized the mice cages prior to the experiment. At the end of the anxiety tests, each mouse was weighed and its surface area calculated by measuring the amount of pixels per mouse frame in the recording of the elevated maze. Table 2 indicates the results for NTG and TG mice for average body weight, behaviour on the EPM and in the LD. We observed no significant differences in body weight between FNTG and FTG or MNTG and MTG mice.

**Table 2:** Overview of differences between trangenic and nontransgenic mice in body weight and behaviour on the EPM and in the LD box.

	**Overall**			**Male**			**Female**		
**General**	**NTG**	**TG**	**Diff**	**NTG**	**TG**	**Diff**	**NTG**	**TG**	**Diff**

Population Size	61	48		29	20		32	28	
Average Weigth (g)	23.31	22.46	-0.85	25.34	25.45	0.11	21.47	20.32	-1.15
Stdev weigth (g)	1.93	1.81	-0.12	1.85	2.11	2.81	2.00	1.59	2.55
Surface area (px/ms)	857.22	849.92	-7.30	907.00	910.00	3.00	812.11	807.00	-5.11

**Elevated plus maze**									

**# Head dips**	7.67	11.17	3.49	8.97	8.10	-0.87	6.50	13.36	6.86
Stdev	1.48	2.02	2.50	1.19	1.15	1.66	0.93	3.34	3.47
T-test TG-NTG	0.06			0.31			0.03		
**Time open (%)**	9.61	13.38	3.77	12.07	9.68	-2.40	7.38	16.02	8.65
Stdev (%)	0.03	0.04	0.04	4.08	4.89	6.37	3.34	5.47	6.42
T-test TG-NTG	0.21			0.35			0.09		
**Time closed (%)**	87.24	84.07	-3.17	85.30	88.53	3.23	89.00	80.89	-8.11
Stdev (%)	0.03	0.04	0.05	4.07	4.81	6.30	3.52	5.48	6.51
T-test TG-NTG	0.25			0.31			0.11		
**Time center (%)**	3.15	2.55	-0.60	2.63	1.80	-0.83	3.62	3.09	-0.53
Stdev (%)	0.01	0.00	0.01	0.71	0.26	0.76	1.18	0.00	1.18
T-test TG-NTG	0.24			0.14			0.34		
**#entries open**	2.55	2.63	0.08	2.57	2.55	-0.02	2.53	2.68	0.15
Stdev	0.21	0.28	0.35	0.29	0.33	0.44	0.32	0.43	0.53
T-test TG-NTG	0.41			0.49			0.39		
**#entries closed**	8.63	8.65	0.01	8.27	9.30	1.03	8.97	8.18	-0.79
Stdev	0.53	0.63	0.82	0.75	0.85	1.13	0.75	0.85	1.14
T-test TG-NTG	0.49			0.19			0.24		

**Light dark box**									

**Transitions**	15.19	15.73	0.54	13.53	16.45	2.92	16.70	15.22	-1.48
Stdev	0.76	0.89	1.17	1.04	1.28	0.23	1.10	1.24	0.14
T-test TG-NTG	0.35			0.11			0.26		
**%time open**	11.93	14.10	2.17	12.29	14.22	1.94	11.61	14.02	2.41
Stdev	0.01	0.02	0.02	1.37	2.45	1.08	1.24	3.18	1.94
T-test TG-NTG	0.16			0.25			0.24		
**%time closed**	88.07	85.90	-2.17	87.71	85.78	-1.94	88.39	85.98	-2.41
Stdev	0.01	0.02	0.02	1.37	2.45	1.08	1.24	3.18	1.94
T-test TG-NTG	0.16			0.25			0.24		
**Mouse activity (px/ms. fr)**	286.20	301.50	15.30	253.51	300.48	46.97	315.83	302.23	-13.6
Stdev	20.58	25.76	32.97	28.08	39.99	11.91	29.45	34.32	4.87
T-test TG-NTG	0.30			0.16			0.38		

FNTG: Female non-transgenic littermates FTG: Female transgenic mice; MNTG: Male non-transgenic littermates; MTG: Male transgenic mice; StDev: standard deviation. A one tailed T-test was performed to assess the significance of each measured difference. When the variation of the two groups was the same we relied on the homoscedastic T-test, otherwise we used the heteroscedastic T-test. Mouse activity was measured in pixels per mouse frame (px/ms.fr).

We performed the EPM first, because of its high sensitivity towards previous experience [18,19]. Traditionally, the nose dips on the open arms, the time spent in the open/closed compartments and the number of entries into open/closed arms are recorded. Table 2 illustrates these parameters for each gender in TG and NTG mice. In our case, we find on average 6.86 (p = 0.03) more head dips in FTG mice than in FNTG littermates, whereas there is no significant difference in head dips between MTG and MNTG mice. The MK5_*L*337*A *_female mice spend on average 8.65% (p = 0.09) more time in the open arms compared to the FNTG, whereas there is no significant difference between MNTG and MTG mice. We found no statistically significant differences between FNTG, FTG, MNTG and MTG mice on the number of entries into the open arm of the EPM.

**Figure 3:**
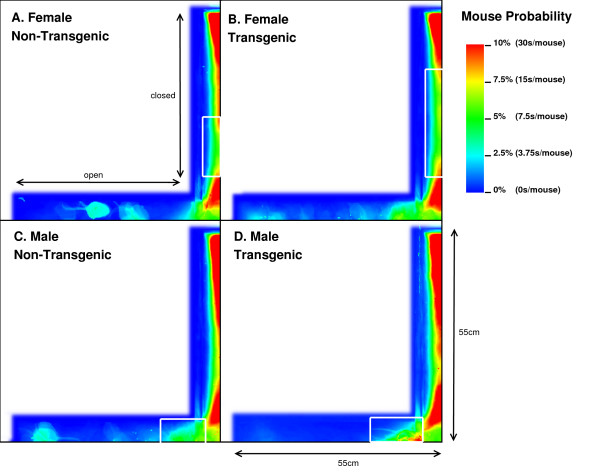
Overview of the mouse presence probability on the EPM over a 5 minute period. Each graph represents for each gender in a specific genetic background, where on the maze the mice are present most. Increased presence is indicated in red, whereas blue reflects a lower probability to find the mouse in that area. The X-axis and Y-axis illustrate the superposition of the two open and closed arms respectively. The sharp mouse contour in the middle of the open arm in graph A and at the end of the open arm in graph C illustrates that one mouse resided there for a long period. This mouse is considered an outlier, since it does not represent the rest of that mouse population. Since this graph is a superposition of each population, a more representative sample would be indicated by more blurry edges.

In support of these findings, our image analysis indicates that FTG mice explored further into the open arm compared to NTG littermates (Figures 3A and 3B). This figure also illustrates a FNTG mouse shape in the middle of the open arm. Since our imaging technique uses superposition of the images to acquire a general distribution, the more general the behaviour is, the more blurrier shapes become. Therefore, the sharp contours of the mouse in Figure 3A indicate it is an outlier. This contrasts to Figure 3B, where the mouse presence is more extended into the open arm and the shape is more blurred. We did not remove the outlier because its presence or absence does not influence the end result. We noticed a similar, though slightly more blurred shape in the MNTG mouse at the end of the open arm in Figure 3C. Aside from this animal, the mouse presence probability between MNTG and MTG is very similar. In Figure 3C and 3D, the white boxes near the beginning of the open arm indicate a similar contribution of the mouse presence in MNTG and MTG mice, with the red area in Figure 3D compensating for the more extended area in Figure 3C. However, we can distinguish that MNTG ventured further into the open arm compared to MTG mice.

Taken together, for NTG and TG mice, we found no differences in the number of entries into the open arms, but an increased number of head dips and time spent on the open arm of the EPM for TG compared to NTG mice. When subdivided into genders, we found that FTG mice displayed an increased amount of head dips on the open arm and resided longer in the open arm compared to littermate controls, but did not differ in the number of open arm entries. Additionally, our image analysis allowed us to detect that FTG mice also ventured further into the open arm compared to FNTG controls. MNTG and MTG mice did not differ in open arm head dips or time spent in the open arm, but MNTG explored further into the open arm compared to MTG. These results indicate a difference between FTG and FNTG in open arm exploration on the EPM, in favour of FTG being less anxious. It also demonstrates a gender difference between female and male mice in their exploration of the open arm which would have escaped us if we had not distinguished between gender.

### Mouse presence probability and activity in the closed arm on the EPM

**Figure 4:**
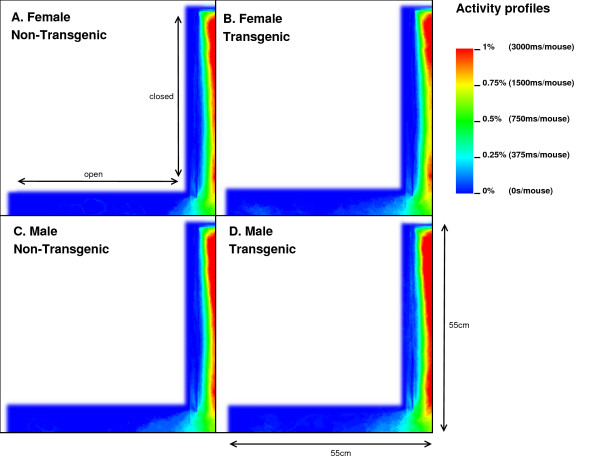
Overview of the mouse activity profiles in the EPM. Each graph represents, the activity in the open and closed arms for TNFG (panel A), FTG (panel B), MNTG (panel C) and MTG (panel D). Mouse activity is indicated in colour with red being highest activity, whereas blue reflects low mouse activity in that area. The X-axis and Y-axis illustrate the superposition of the two open and closed arms respectively.

Analysis of the total entries, the entries into the closed arms and rearings may give an indication on the activity of the mice [20]. As Table 2 shows, we observed a 3.17% (p = 0.05) reduction in time spent in the closed arm between TG and NTG mice. This difference accounts for the increased percentage of time spent in the open arm of the EPM by FTG. For FNTG and FTG mice, the centre time did not differ significantly, whereas MTG spent less time there than MNTG. The mouse presence probabilities in the closed arms (Figure 3) revealed no overt differences between MNTG and MTG mice (Figure 3C and 3D), but as the white box indicates, they did display a difference between FTG and FNTG mice (Figure 3A and 3B). Apparently, FTG spent less time in exploring the centre of the closed arm than FNTG mice. They may compensate the lack of exploration in the centre by their presence near the extreme end of the closed arm or near the centre of the maze (compare Figure 3A and 3B). Our activity profiles in Figure 4 divulged no obvious differences in activity between FTG, FNTG, MNTG and MTG mice and confirmed the results from the time spent and entries into the arms of the maze.

### Detailed analysis of the mouse presence probability and activity profiles on the elevated plus maze

**Figure 5:**
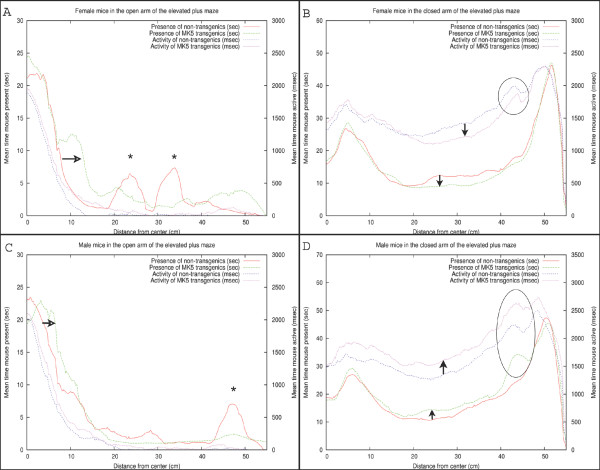
A summary of the mouse probability and activity profiles on the EPM. The X-axis on each graph indicates the distance from the centre area to the extreme end of an arm in cm. In addition, we use two Y-axes for each graph: the left Y-axis displays the presence of the mouse (mouse presence probability function) in seconds in a particular area (X-axis), the right Y-axis displays the time the mouse is active (mouse activity profile) in milliseconds in a particular area (X-axis). The asterisks in graphs A and C indicate outliers, while the arrows in graphs B and D indicate the difference from FNTG mice to FTG mice and from MNTG to MTG in presence and activity profiles. The black ellipses indicate the effect of the presence of a shadow in the closed arm on mouse activity and presence. The horizontal arrows in graphs A and C indicate the difference between NTG and TG mice.

Next, we monitored the mouse probability and activity profiles per gender in the presence or absence of the MK5_*L*337*A *_transgene. As illustrated in Figure 5A, the FTG explored the open arm of the maze more extensively than the FNTG mice (compare green and red curves). This conduct is indicated by the horizontal arrow and an increased area under the curve over almost the entire open arm in Figure 5. The two asterisks correspond with the two outliers on the open arm (Figure 5A). Both FNTG and FTG mice seem to display similar activity near the beginning of the open arm, but the activity of the FTG mice is higher over the rest of the open arm (compare blue and magenta curves in Figure 5A).

As shown in the previous section, FNTG mice displayed a higher activity from the middle of the closed arm towards the end, as indicated by a smaller white box in Figures 3A and 3B. Similarly, in Figure 5B, we see that FNTG mice display a higher activity from the middle of the closed arm to the end (vertical downward arrow). The activity is accompanied by a higher mouse presence in the middle of the closed arm, but resembles that of FTG mice near the end of the closed arm. This indicates that FNTG are more active and more present in the centre area of the closed arm compared to the FTG mice. The black ellipse reflects that mice actively investigate a small shadow cast at the end of the closed arm, but this exploration does not last long enough to influence the total mouse presence.

The MTG mice are more present in the first part of the open arm compared to MNTG mice as indicated by Figure 3C and 3D and by the horizontal open arrow in Figure 5C. However, the remainder of the mouse presence probability of MTG resembles that of MNTG, as illustrated in Figure 5C.

The results in Figure 5D showed that MTG mice resided more in the centre area of the closed arm than MNTG mice (upward arrow between red and green curves). The exact opposite occurred in FTG versus FNTG mice in Figure 5B (downward arrow between red and green curves). In addition, the exploration of the shadow area near the end of one of the closed arms resulted in an increased presence of MTG mice, but not of MNTG, FTG or FNTG mice (compare red and green curves in the black ellipse in Figures 5B and 5D). We observed a similar distribution for the activity profiles in the closed arm (blue and magenta curves in Figures 5B and 5D). Here, MTG mice are more active over the entire closed arm, compared to MNTG mice (upward arrow in Figure 5D). This contrasts to FTG mice which are less active in the centre and near the end of the closed arm compared to FNTG mice (downward arrow in Figure 5). Activity profiles for the shadow area, indicated by the black ellipse (Figures 5B and 5D), revealed increased activity in FNTG and MTG mice, compared to FTG and MNTG mice respectively. Table 3 illustrates how strongly complementary these results are in terms of gender and influence of the transgene.

**Table 3:** Summary of the results.

Parameters	Female	Male	Effect on	Opposite
**EPM**				

Head dips	6.86	-0.87	Female	Yes
% time open	8.65	-2.4	Female	Yes
% time closed	-8.11	3.23	Female	Yes

**LD**				

Transitions	-1.48	2.92	Male	Yes
% time open	2.41	1.94	Female	No
% time closed	-2.41	-1.94	Female	No
Activity light	-13.6	46.97	Male	Yes

We compared TG mice to NTG mice per gender. A negative sign that indicates TG mice display a decreased response for a particular parameter compared to NTG mice. "Effect on" displays which gender is most affected, while "Opposite" indicates whether the behaviour of male mice is opposite to that observed in female mice.

In summary, FTG mice displayed an increased presence in the open arm, and a lower presence and activity in the middle of the closed arm, when compared to FNTG mice. MTG are more present in the first part of the open arm, but resemble the MNTG for the rest of the open arm. In the closed arm, MTG are more present in the middle of and more active over the entire closed arm compared to MNTG mice. They also investigate the shadow area more profoundly, resulting in increased presence and activity.

### Light-dark box

**Figure 6:**
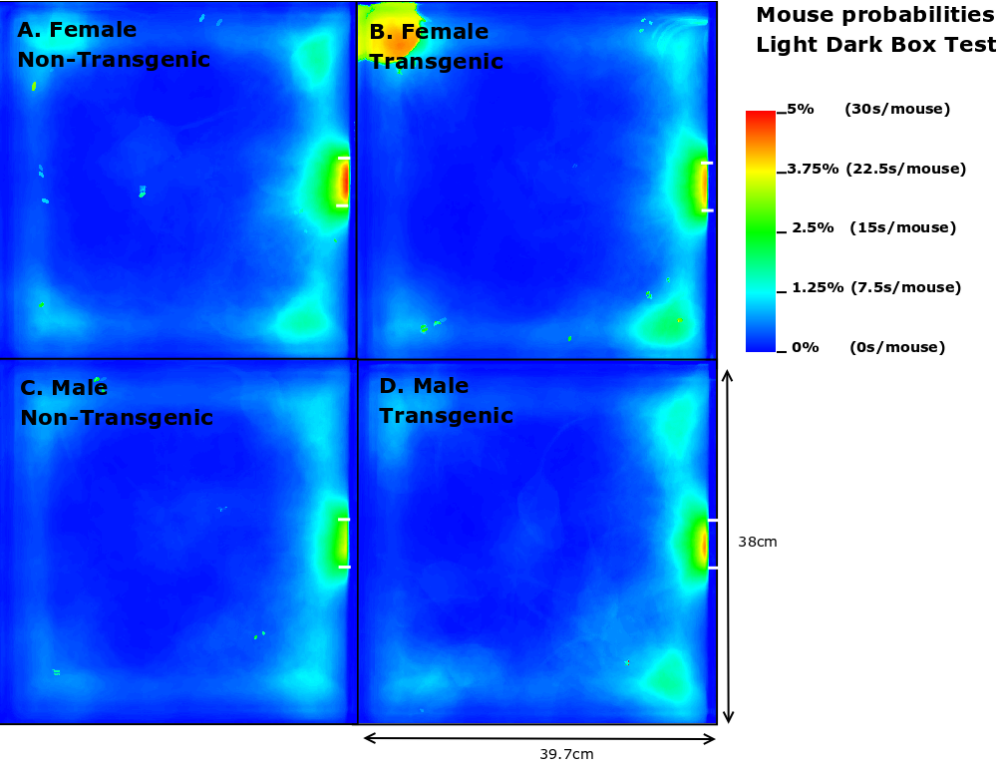
Mouse presence in the light area during the light-dark test. The horizontal white lines in the middle of the right edge illustrate the entrance to the dark compartment. Each mouse was videorecorded for 10 minutes. Different colour grading indicates the duration of the presence by a mouse on a particular spot.

In contrast to the results on the EPM, MTG in the LD box test performed 2.92 times more transitions compared to MNTG mice and they spent 1.94% longer in the open compartment (Table 2). FTG mice also resided longer (2.41%) in the light compartment of the LD, but performed fewer transitions compared to FNTG mice. This confirms our mouse probability profiles (Figure 6) which show that FNTG and MTG mice explored the light compartment more extensively, as indicated by the intense colouring in these regions. For MTG, the increased time spent in the light compartment may be an indirect effect due to increased activity. Indeed, as Table 2 indicated, MTG mice were more active (46.97 pixels/mouse frame) compared to MNTG. FTG mice were less active than FNTG mice (13.6 pixels/mouse frame), but FTG mice explored the light compartment for a longer time period. Table 3 confirms these complementary responses in FTG versus FNTG and MTG versus MNTG. In the mouse presence probability of the FTG mice, we find an outlier in the upper left corner in Figure 6B. This results from a mouse that remained there for almost the entire observation time. However, its removal did not influence the end result.

Taken together, we found that FTG and MTG mice explored the light compartment longer compared to their NTG littermates. However, in contrast to FTG and MNTG mice, FNTG and MTG mice showed a significantly increased activity in the light area. This may indicate that the increased activity in MTG compensates for the increased time spent in the open compartment. It also confirms a reduced anxiousness in FTG mice, because they resided longer in the light compartment, despite decreases in their activity.

## Discussion

Since the constitutive expression of a protein with aberrant function in an animal model may provide insight into the biological functions of this protein we constructed transgenic mice that expressed constitutive active MK5, MK5_*L*337*A*_. MK5_*L*337*A*_ mice were fertile and displayed no obvious physical or morphological differences compared to control littermates. Initial observations from the SHIRPA analysis suggested differences in anxiety-related behaviour. Subsequently, these mice were subjected to two validated anxiety tests: the elevated plus maze and the light-dark box test.

### Difference between males and females

This study revealed more differences between NTG and TG mice when we subdivided both groups per gender. Our data imply that while there exist small differences between NTG and TG mice, these do not necessarily represent the situation per gender in NTG and TG groups. Additionally, we observed opposite reactions between males and females and between NTG and TG mice for several parameters. For example, FNTG mice are more active in the light compartment of the LD compared to FTG mice, whereas MTG mice also displayed increased activity compared to MNTG mice (see Tables 2 and 3).

Since the endogenous MK5 gene resides on murine chromosome 5 and the transgenic MK5_*L*337*A *_gene is inherited in an autosomal manner (our unpublished results), it is unlikely that we can attribute the observed gender differences to a sex-linked transgene. However, it remains possible that MK5 targets a substrate involved in regulatory processes that differ between genders. For example, our microarray data suggest that upon increased expression of MK5_*L*337*A *_DAZAP2 becomes downregulated. The function of DAZ-associated protein2 (DAZAP2) remains unelucidated but studies on its family member DAZAP1 indicate that the gene is expressed during sperm maturation, where it shuttles between the nucleus and the cytoplasm during the different stages [21-23]. In addition, DAZ itself is involved in the repression of genes that induce female development and is exclusively found on the Y-chromosome and hence essential for male development. This may partially explain why MTG mice resemble FNTG in behaviour in the closed arm of the EPM, but it does not explain why MNTG mice resemble FTG under these circumstances.

In addition, although MTG mice seem less anxious in the LD, the EPM data do not support such a behaviour. The discrepancy in behaviour between the results of the two tests could be attributed to slightly different forms of anxiety tested in the EPM versus the LD. However, a more likely reason for this difference lays in the increased activity of the MTG mice in the LD test. Since this test does not automatically compensate for differences in activity, as the EPM does, an open field test could help determine whether MTG presence in the open compartment is linked to increased activity. On the other hand, we did not observe any differences in the number of entries, or an increased activity on the open arm on the EPM for MTG mice (Figure 5C) [14].

### Anxiety-related behavioural processes

Our results indicate that FTG mice on the EPM display more head dips on the open arm and reside there longer compared to FNTG, MNTG and MTG mice (see Table 2). A similar tendency occurs with the MTG mice in the light compartment of the LD. This behaviour, the increased presence in the light compartment, implies reduced anxiousness [20,24]. Therefore, these findings could indicate a role for MK5 in reduced anxiety-related processes that may affect females more than males.

Anxiety-related differences on the EPM and in the LD have been described in other mouse models, particularly in relation to serotonin (5HT) signalling and availability. A recent report by Drapier *et al *revealed that inhibition of 5-HT and noradrenaline by pharmacological compounds resulted in decreased time spent in the open arm of the EPM or decreased exploration in the open arm, only when 5-HT reuptake was inhibited [25]. These results confirm earlier studies with pharmacological compounds against the SERT or KO models of the gene for SERT, which all augmented anxious behaviour, regardless of postnatal stress [13,26-28].

Anxiety-related processes in relation to gender have also been described in a double KO mouse model of the serotonin transporter (*S*ERT) and the brain-derived growth factor receptor (*B*DNFR), referred to as *sb *mice. Male *sb *mice show increased anxious behaviour, indicated by less time spent in and less frequent entry into the open arm, while the mutation did not affect anxiousness in female *sb *mice [28]. Although our results indicated no equal division in gender responses on the EPM. Another explanation for our results may lie in gender differences in thresholds. For example, in 5-HT_1*A *_KO mice, male KO mice displayed an increased impulsiveness, as measured by a shorter centre time and shorter distances traveled within the centre. Female KO mice remained unaffected and the researchers attributed these effects to differences in threshold levels between the genders [20]. Similarly, expression of constitutive active MK5 may lower the threshold for female mice in exploration of the maze, whereas males may be more resistant.

In a KO mouse model of SERT alone, administration of 5HT_1*A *_receptor antagonists produced a gender-independent anxiolytic effect on the EPM thereby indicating subtle but persistent perturbations in 5HT homeostasis [29]. Since we also observe rather small but significant and persistent perturbations, this may imply a role for MK5 in 5HT regulatory mechanisms. Even more, the upstream activator for MK5, p38, becomes activated by stress stimuli and stimuli that activate the A_3 _adenosine receptor in a PKG dependent and independent way. This p38 activation results in increased activity of SERT and thereby causes increased reuptake of 5HT [30,31]. If MK5 intervenes in this signalling cascade it could do so by phosphorylating cytoskeletal proteins involved in transport of the SERT from vesicles to the membrane, or proteins associated with the SERT, thereby affecting their affinity for the transporter. Further experiments are necessary to confirm a role for MK5 in anxiety-related processes and if such a role exists, MK5 could become an interesting target for drug exploration.

As to date, there exist few reports on the involvement of MAPKs in anxiety, and none on the involvement of MAPKAPKs. However MAPKs do play a role in fear-related processes [32-34]. It would be interesting to explore further whether our observations comprise a fear-related component or not.

### Activity

In contrast to the data from the LD and the closed arm of the EPM, we did not observe an increased activity of FNTG and MTG mice on the open arms of the maze (Figure 5B and 5D and Table 2). One might argue that the lower activity due to the lower presence for FTG in the closed arm of the maze compensates for the increased presence and activity in the open arm. However, FTG are equally active as FNTG in the open arm of the maze (Figure 5A). Differences in locomotion can be often attributed to alterations in dopaminergic systems. This is illustrated by the KO model for tyrosine hydroxylase (TH), the enzyme catalyzing the rate-limiting step during the synthesis of dopamine, where mice exhibit deficiencies in locomotor activity [35]. MK5 has been shown to phosphorylate TH *in vitro *[36-38]. However, since no *in vivo *role has been documented, we require further exploration of this pathway to be able to couple MK5 to the regulation of dopamine synthesis and locomotion.

Other mouse models that differ in locomotor activity comprise mice that contain a deficient G-protein coupled inwardly rectifying potassium channel (GIRK) or an ATP-dependent K^+^channel (KATP+
 MathType@MTEF@5@5@+=feaafiart1ev1aaatCvAUfKttLearuWrP9MDH5MBPbIqV92AaeXatLxBI9gBaebbnrfifHhDYfgasaacPC6xNi=xH8viVGI8Gi=hEeeu0xXdbba9frFj0xb9qqpG0dXdb9aspeI8k8fiI+fsY=rqGqVepae9pg0db9vqaiVgFr0xfr=xfr=xc9adbaqaaeGacaGaaiaabeqaaeqabiWaaaGcbaGaee4saS0aa0baaSqaaiabdgeabjabdsfaujabdcfaqbqaaiabgUcaRaaaaaa@3164@). In the KATP+
 MathType@MTEF@5@5@+=feaafiart1ev1aaatCvAUfKttLearuWrP9MDH5MBPbIqV92AaeXatLxBI9gBaebbnrfifHhDYfgasaacPC6xNi=xH8viVGI8Gi=hEeeu0xXdbba9frFj0xb9qqpG0dXdb9aspeI8k8fiI+fsY=rqGqVepae9pg0db9vqaiVgFr0xfr=xfr=xc9adbaqaaeGacaGaaiaabeqaaeqabiWaaaGcbaGaee4saS0aa0baaSqaaiabdgeabjabdsfaujabdcfaqbqaaiabgUcaRaaaaaa@3164@ KO model, the mice resided less in the centre and closed arm of the EPM, and longer in the open arm. In addition, they showed increased activity in the home cage but reduced activity in novel environments [39,40]. For GIRK deficiency, the mice also displayed decreased anxiety, but in addition, these mice displayed increased locomotion [39]. These studies suggest the possibility to alter both anxiety-related processes and locomotor activity at the same time. This is interesting for our results since we also observed differences in activity and anxiety. Even more, our microarray analysis reported that expression of MK5_*L*337*A *_altered the expression of solute carriers and KATP+
 MathType@MTEF@5@5@+=feaafiart1ev1aaatCvAUfKttLearuWrP9MDH5MBPbIqV92AaeXatLxBI9gBaebbnrfifHhDYfgasaacPC6xNi=xH8viVGI8Gi=hEeeu0xXdbba9frFj0xb9qqpG0dXdb9aspeI8k8fiI+fsY=rqGqVepae9pg0db9vqaiVgFr0xfr=xfr=xc9adbaqaaeGacaGaaiaabeqaaeqabiWaaaGcbaGaee4saS0aa0baaSqaaiabdgeabjabdsfaujabdcfaqbqaaiabgUcaRaaaaaa@3164@ channels. It will be interesting to explore these options for MK5 as a regulator in neurological processes.

## Conclusion

In this study we observed gender differences in the behaviour of transgenic mice expressing a constitutive active form of MK5. We found that female transgenic mice (FTG) displayed less anxious behaviour on the elevated plus maze and in the light-dark test compared to their littermate controls (FNTG). Male transgenic mice displayed similar anxiety profiles to male non-transgenic mice, but displayed increased activity in the closed arm of the maze. A behaviour that contrasted to FTG versus FNTG mice. In addition, our image analysis method also allowed us to detect that FTG explored further into the open arm on the elevated plus maze. Since both anxiety-related processes and locomotor activity can be regulated by differences in neurotransmitter metabolism, this may indicate for the first time a role for a MAPKAPK in such a process.

## Competing interests

The author(s) declare that they have no competing interests.

## Authors' contributions

NG constructed the vector for transgene delivery, checked expression levels, performed genotyping, performed preliminary SHIRPA measurements, sacrificed and autopsied the mice, performed the elevated plus maze and light dark tests, helped designing the experiments and wrote the manuscript. WVB helped setting up the anxiety tests, provided the cameras, performed the data and statistical analysis and helped writing the manuscript. UM conceived of the transgenic mouse experiment, participated in the design and coordination of the experiments, helped writing the manuscript. All authors read and approved the final manuscript.

## References

[B1] Einat H, Yuan P, Gould TD, Li J, Du J, Zhang L, Manji HK, Chen G (2003). The role of the extracellular signal-regulated kinase signaling pathway in mood modulation. J Neurosci.

[B2] Mourin CI, Huot J (2004). Recent advances in stress signaling in cancer. Cancer Res.

[B3] Roux PP, Blenis J (2004). ERK and p38 MAPK-activated protein kinases: a family of protein kinases with diverse biological functions. Microbiol Mol Biol Rev.

[B4] Sweat JD (2001). The neuronal MAP kinase cascade: a biochemical signal integration system subserving synaptic plasticity and memory. J Neurochem.

[B5] Gaestel M (2006). MAPKAP kinases – MKs- two's company, three's a crowd. Nat Rev Mol Cell Biol.

[B6] Schumacher S, Laas K, KAnt S, shi Y, Kotlyarov A, Gaestel M (2004). Scaffolding by ERK3 regulates MK5 in development. EMBO J.

[B7] Shi Y, Kotlyarov A, Laas K, Gruber AD, Butt E, Marcus K, Meyer HE, Friedrich A, Volk HD, Gaestel M (2003). Elimination of protein kinases MK5/PRAK activity by targeted homologous recombination. Mol Cell Biol.

[B8] Sun P, Yoshizuka N, New L, Moser BA, Li Y, Liao R, Xie C, Chen J, Deng Q, Yamout M, Dong MQ, Frangou CG, YatesIII JR, Wright PE, Han J (2007). PRAK is essential for ras-induced senescence and tumor suppression. Cell.

[B9] Gerits N, Kostenko S, Moens U (2007). In vivo functions of mitogen-activated protein kinases: conclusions from knock-in and knock-out mice. Transgenic Res.

[B10] Seternes OM, Johansen B, Hegge B, Johannessen M, Keyse SM, Moens U (2002). Both binding and activation of p38 mitogen-activated protein kinase (MAPK) play essential roles in regulation of the nucleocytoplasmic distribution of MAPK-activated protein kinase 5 by cellular stress. Mol Cell Biol.

[B11] Rogers D, Fisher E, Brown S, Peters J, Hunter A, Martin J (1997). SHIRPA protocol: behavioral and functional analysis of mouse phenotype assessment. Mamm Genome.

[B12] Crawley JN (2007). What's wrong with my mouse: behavioral phenotyping of transgenic and knockout mice.

[B13] Holmes A, Li Q, Murphy D, Gold E, Crawley J (2003). Abnormal anxiety-related behavior in serotonin transporter null mutant mice: the influence of genetic background. Genes Brain Behav.

[B14] Bourin M, Hascoët M (2003). The mouse light/dark box test. Eur J Pharmacol.

[B15] Press WH, Teulkolsky SA, Vetterling WT, Flannery BP (2002). Numerical recipes in C++.

[B16] Yates F (1934). Contingency table involving small numbers and the *χ*^2 ^test. J R Stat Soc.

[B17] Killeen P (2005). An alternative to null-hypothesis significance tests. Psychol Sci.

[B18] Holmes A, Rodgers R (1999). Influence of spatial and temporal manipulations on the anxiolytic efficacy of chlordiazepoxide in mice previously exposed to the elevated plus-maze. Neurosci Biobehav Rev.

[B19] Izidio G, Spricigo L, Ramos A (2005). Genetic differences in the elevated plus-maze persist after first exposure of inbred rats to the test apparatus. Behav Processes.

[B20] Ramboz S, Oosting R, Amara DA, Kung HF, Blier P, Mendelsohn M, Mann JJ, Brunner D, Hen R (1998). Serotonin receptor 1A knockout: an animal model of anxiety-related disorder. Proc Natl Acad Sci USA.

[B21] Dai T, Vera Y, Salido EC, Yen PH (2001). Characterization of the mouse DazapI gene encoding an RNA-binding protein that interacts with infertility factors DAZ and DAZL. BMC Genomics.

[B22] Morton S, Yang HT, Moleleki N, Campbell DG, Cohen P, Rousseau S (2006). Phosphorylation of the ARE-binding protein DAZAP1 by ERK2 induces its dissociation from DAZ. Biochem J.

[B23] Vera Y, Dai T, Hikim APS, Lue Y, Salido EC, Swerdloff RS, Yen PH (2002). Deleted in Azoospermia Associated Protein 1 shuttles between nucleus and cytoplasm during normal germ cell maturation. J Androl.

[B24] Slotten HA, Kalinichev M, Hagan JJ, Marsden CA, Fone KCF (2006). Long-lasting changes in behavioural and neuroendocrine indices in the rat following neonatal maternal separation: gender-dependent effects. Brain Res.

[B25] Drapier D, Bentue-Ferrer D, Laviolle B, Millet B, Allain H, Bourin M, Reymann J (2007). Effects of acute fluoxetine, paroxetine and desipramine on rats tested on the eleveated plus-maze. Behav Brain Res.

[B26] Carroll JC, Boyce-Rustay JM, Millstein R, Yang R, Wiedholz LM, Murphy DL, Holmes A (2007). Effects of mild early life stress on abnormal emotion-related behaviors in 5-HTT knockout mice. Behav Genet.

[B27] Kurt M, Arik A, Celik S (2000). The effects of sertraline and fluoxetine on anxiety in the elevated plus-maze test in mice. J Basic Clin Physiol Pharmacol.

[B28] Ren-Patterson RF, Cochran LW, Holmes A, Lesch KP, Lu B, Murphy DL (2006). Gender-dependent modulation of brain monoamines and anxiety-like behaviors in mice with genetic serotonin transporter and BDNF deficiencies. Cell Mol Neurobiol.

[B29] Lesch K, Mössner R (2006). Inactivation of 5HT transport in mice: modeling altered 5HT homeostasis implicated in emotional dysfunction, affective disorders and somatic syndromes. Handb Exp Pharmacol.

[B30] Zhu CB, Carneiro AM, Dostmann WR, Hewlett WA, Blakely RD (2005). p38 MAPK activation elevates serotonin transport activity via a trafficking-independent, protein phosphatase 2A-dependent process. J Biol Chem.

[B31] Zhu CB, Hewlett WA, Feoktistov I, Biaggioni I, Blakely RD (2004). Adenosine receptor, protein kinase G, and p38 mitogen-activated protein kinase-dependent up-regulation of serotonin transporters involves both transporter trafficking and activation. Mol Pharm.

[B32] Davis M (2002). Role of NMDA receptors and MAP kinase in the amygdala in extinction of fear: clinical implications for exposure therapy. Eur J Neurosci.

[B33] Sindreu CB, Scheiner ZS, Storm DR (2007). Ca^2+^-stimulated adenylyl cyclases regulate ERK-dependent activation of MSK1 during fear conditioning. Neuron.

[B34] Szapiro G, Vienna MR, McGaugh JL, Medina JH, Izquierdo I (2003). The role of NMDA glutamate receptors, PKA, MAPK, and CAMKII in the hippocampus in extinction of conditioned fear. Hippocampus.

[B35] Bornstein S, Tian H, Haidan A, Böttner A, Hiroi N, Eisenhofer G, McCann S, Chrousos G, Roffler-Tarlov S (2000). Deletion of tyrosine hydroxylase gene reveals functional interdependence of adrenocortical and chromaffin cell system in vivo.. Proc Natl Acad Sci USA.

[B36] Dunkley PR, Bobrovskaya L, Graham ME, von Nagy-Felsobuki EI, Dickson PW (2004). Tyrosine hydroxylase phosphorylation: regulation and consequences. J Neurochem.

[B37] Toska K, Kleppe R, Armstrong CG, Morrice NA, Cohen P, Haavik J (2002). Regulation of tyrosine hydroxylase by stress-activated protein kinases. J Neurochem.

[B38] Toska K, Kleppe R, Cohen P, Haavik J (2002). Phosphorylation of tyrosine hydroxylase in isolated mice adrenal glands. Ann N Y Acad Sci.

[B39] Blednov YA, Stoffel M, Chang S, Harris RA (2001). Potassium channels as targets for ethanol: studies of G-protein-coupled inwardly rectifying potassium channel 2 (GIRK2) null mutant mice. J Pharmacol Exp Ther.

[B40] Deacon R, Brook R, Meyer D, OHaeckel, Ashcroft F, Miki T, Seino S, Liss B (2006). Behavioral phenotyping of mice lacking the k_*ATP *_channel subunit Kir6.2. Physiology and Behavior.

